# Role of Oct3/4 in Cervical Cancer Tumorigenesis

**DOI:** 10.3389/fonc.2020.00247

**Published:** 2020-03-11

**Authors:** Sayuri Itzel Clemente-Periván, Yazmín Gómez-Gómez, Marco Antonio Leyva-Vázquez, Alfredo Lagunas-Martínez, Jorge Organista-Nava, Berenice Illades-Aguiar

**Affiliations:** ^1^Laboratorio de Biomedicina Molecular, Facultad de Ciencias Químico Biológicas, Universidad Autónoma de Guerrero, Chilpancingo, Mexico; ^2^Centro de Investigación sobre Enfermedades Infecciosas, Instituto Nacional de Salud Pública, Cuernavaca, Mexico

**Keywords:** cervical cancer, cancer stem cells, OCT3/4, proliferation, self-renewal

## Abstract

Cervical cancer (CC) is the fourth most common type of cancer that affects women. Compared to other types of cancer, CC has a high mortality rate in women worldwide. Several factors contribute to the development of CC, but persistent high-risk human papillomavirus infection is the main etiologic agent associated with the development of CC. Moreover, several studies reported that alterations in the expression of transcription factors present in a small subpopulation of cells within tumors called cancer stem cells (CSCs), which contribute to the development of CC by promoting tumorigenicity and metastasis. These transcription factors affect self-renewal and maintenance of pluripotency and differentiation in stem cells. *OCT3/4* belongs to the family of transcription factors with the POU domain. It consists of five exons and can be edited by alternative splicing into three main transcripts: OCT3/4A, OCT3/4B, and OCT3/4B1. The OCT3/4 expression in CSCs promotes carcinogenesis and the development of malignant tumors, and the loss of expression leads to the loss of self-renewal and proliferation and favors apoptosis. This review describes the main roles of OCT3/4 in CC and its importance in several biological processes that contribute to the development of CC and may serve as molecular targets to improve prognosis of CC.

## Introduction

Cervical cancer (CC) is a worldwide public health problem. In 2018, there were an estimated 569,847 new cases and 311,365 deaths due to CC. It occupies the fourth place in women's, among all cancers with respect to incidence and mortality worldwide (1). Cervical cancer is more common in developing countries, where 85% of cases were observed, than in the rest of the world (2).

Cervical cancer is a multifactorial disease. Some of the factors associated with CC included the use of oral contraceptives for more than 5 years, multiparity, socioeconomic status, obesity, pregnancy, and sexual activity at an early age (3). Persistent human papillomavirus (HPV) infection, mainly HPV types 16 and 18, is the most important factor, which is related to 99.7% of cervical squamous cell cancer cases worldwide (4). Although most HPV infections are transient and eliminated by the immune system (5), factors such as the immune status, coinfection, parity, and smoking can lead to a chronic high-risk (HR) HPV infection that favors the development of preneoplastic lesions (6). In addition, recent studies revealed the importance of cancer stem cells (CSCs) in the development of resistance, metastases, and tumorigenicity of cancer (7–9).

The CSCs and embryonic stem cells (ESCs) share similar characteristics such as self-renewal, unlimited proliferation, and the ability to maintain undifferentiated status. These characteristics are maintained by stem cell markers such as SOX2, NANOG, and OCT3/4, the latter belonging to the family of transcription factors with the POU domain (10). Recently, it has been indicated that these stem cell markers are highly expressed during the tumorigenesis process (11); however, their role in the processes that favor the development of CC remains unclear. The present mini review discusses the importance of OCT3/4 in acquisition of CSCs characteristics in CC. This mini review provides a broad view regarding OCT3/4, a marker that might be an appropriate target for therapy to improve the treatment of CC.

## OCT3/4 and Its Role in CC

### OCT3/4 and Stem Cells

Stem cells are cells that divide symmetrically to give rise to daughter cells for self-renewal and amplification, as well as asymmetrically to produce specific and differentiated lineages (12–14).

OCT3/4 (or POU5F1) is a transcription factor that binds as an octamer and is a key regulator of pluripotency, differentiation, and self-renewal in ESCs (15). It was first reported in 1989 (16), and its expression has been observed in ovulated oocytes, early preimplantation embryos, primitive ectoderm, the inner cell mass, ESCs, embryonic germ cells, and embryonic carcinoma cells but not in their differentiated daughters (17, 18).

The *OCT3/4* gene is located on chromosome 6, consists of five exons, and can be edited by alternative splicing into three main transcripts: OCT3/4A, OCT3/4B (19), and OCT3/4B1 (20), and generate four proteins: OCT3/4A, OCT3/4B-190, OCT4B-265, and OCT3/4B-164. OCT3/4A and OCT3/4B/B1 are functionally and structurally divided into an N-terminal transcriptional activation domain, a central POU domain, and a C-terminal cell-type–specific transactivation domain (21). Additionally, new spliced variants of OCT4 have been detected, such as OCT4B2 (22), OCT4B3 (23), OCT4B4 (24), OCT4C, and OCT4C1 (25). These new variants have been identified in different cell lines; however, all showed a decrease in their expression by induction of cell differentiation, demonstrating a role similar to the previously reported variants, which are attributed to maintaining undifferentiated state in the cell (23, 24). However, the location of the different OCT3/4 isoforms correlated with their various functions'; unlike OCT3/4A, OCT3/4B is mainly found in the cytoplasm (26). Cauffman et al. (27, 28) analyzed the expression patterns of OCT3/4A and OCT3/4B during human embryogenesis in human ESCs and found that OCT3/4A had significant expression in all embryo nuclei and compact blasts, and OCT3/4B was expressed in the cytoplasm from the four-cell stage. The localization of OCT3/4B suggests that it may play a role in other biological functions such as stress response (29). On the other hand, the cell self-renewal characteristics of OCT3/4 can be attributed to the OCT3/4A isoform (26).

### OCT3/4 and CSCs

Cancer stem cells are defined functionally as a subset of cells that display stemness characteristics, including the ability to asymmetrically divide, resulting in self-renewal of CSCs and the production of heterogeneous populations of cancer cells (30). The CSCs have been isolated in a variety of solid tumors such as breast cancer, glioblastoma, osteosarcoma, prostate cancer, ovarian cancer, gastric cancer, and lung cancer (31). The expression of OCT3/4 plays an important role in the malignant potential of tumor cells and can be detected in different types of tumors, such as human embryonal carcinomas, testicular germ cell tumors, and gliomas (32, 33). The transcription factors SOX2 and OCT3/4 were proposed as biomarkers for cell-type CSCs of cell lines and malignant tissues such as breast cancer (34, 35), human non–small cell lung cancer (11), bladder cancer, colon cancer, prostate cancer (36), and gastric cancer cells (37). Moreover, these transcription factors that confer “stemness” characteristics to the cancer cells contribute to carcinogenesis, tumor metastasis, and poor results (38, 39).

It was shown that CSCs that expressed OCT3/4 have characteristics that confer chemoresistance and radioresistance (40, 41). López et al. (40) characterized a subpopulation of cells with self-renewal capacity in four cancer-derived cell lines (HeLa, SiHa, CaSki, and C-4 I) and found expression of characteristic markers of stem cell, epithelial–mesenchymal transition (EMT), and radioresistance. These data could contribute to the improvement of therapies aimed at cancer patients and reduction in the mortality caused by this disease. It has been observed that OCT3/4 may be a therapeutic target, because the loss of OCT3/4 expression in cells leads to the loss of self-renewal and proliferation capacities, favoring the process of apoptosis CSCs (42). Therefore, conventional treatments along with therapy directed at markers of CSCs (OCT3/4) are a promising treatment option in efforts to eradicate cancer in clinical settings.

### Oct3/4 in CC

**Figure 1:**
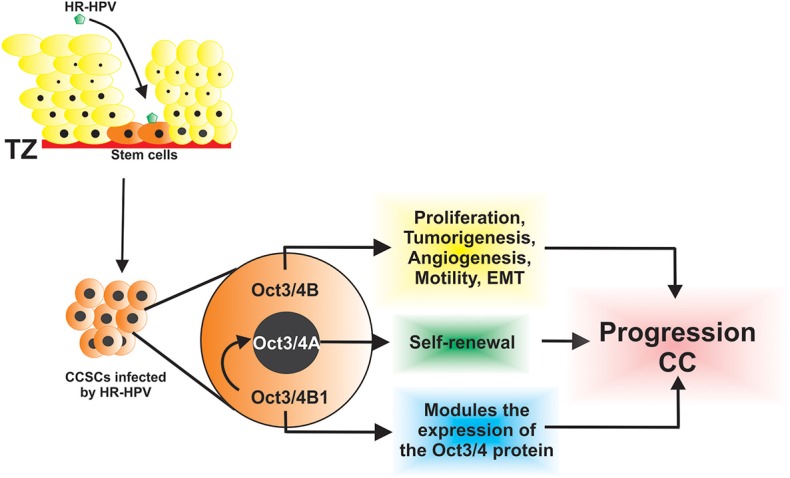
The HR-HPV infects CSCs that express isoforms of Oct3/4 and contributes to the progression of cervical cancer through diverse, key, and biological processes.

High-risk HPV infection targets the cuboidal epithelial cells within the transformation zone that are considered stem cells of the cervical epithelium. The characteristics of these stem cells contribute to the development of CC because they have the capacity for self-renewal and are capable of generating diverse lineages of cancer cells [(10, 43–45); Figure 1]. It was reported that *OCT3/4* can act as an oncogene and trigger cancerous stem cells (46, 47). Several studies were conducted both *in vivo* and *in vitro* to study the role of OCT3/4 in CC. The expression of OCT3/4 was investigated in patients with CC by immunohistochemistry, and it was high in premalignant and malignant cervical tumors (38). Similarly, OCT3/4 expression was strongly associated with poor disease-free survival and overall survival (poor prognosis) in patients with CC, which suggests that OCT3/4 expression is a possible marker for this type of cancer (48).

In samples with CC, it was observed that the expression of OCT3/4 increases according to the degree of injury, and this expression was higher in the nucleus than in the cytoplasm (38). High-risk HPV-positive cell lines such as HeLa and CaSki have higher expression of OCT3/4 in the nucleus than in the cytoplasm, and consequently, these cell lines have higher colony formation capacity than the C-33 A cell line (HPV negative) in which OCT3/4 expression was homogeneous in the nucleus and cytoplasm (49). In addition, the active protein of OCT3/4 is mainly located in the nucleus, whereas the nonfunctional protein with respect to the maintenance of stemness characteristics is mainly located in the cytoplasm (26, 28). These data suggest that the HPV can initiate cervical carcinogenesis through the positive regulation of OCT3/4 (49).

On the other hand, little is known about the mechanism of epigenetic regulation on the level of expression of OCT3/4 in CC cells (49). In 2012, Liu et al. (50) observed that the inhibition of an important member of the histone deacetylase (HDAC) family, histone deacetylase 1 (HDAC1) by valproic acid, can promote the transcription of OCT3/4 in C33A cells. It has been reported that there could be an interaction between the E7 oncoprotein of HPV16 and HDAC1, thus causing the dissociation of the complex between HDAC1 and DNMT3A, leading to an increase in the expression of OCT3/4 (50). Interestingly, the inhibition of DNMT3A and HDAC1 with 5-azacitidine (inducer of DNA hypomethylation) and tricostatin A (HDAC inhibitor) leads to a negative regulation of OCT3/4 expression (51, 52). Likewise, it was observed that the inhibition of DNMT with 5-azacitidine leads to a decrease in the expression of OCT3/4 and the negative regulation of proliferation-associated proteins such as cyclin D1 (52). Cyclin D1 is associated with cyclin-dependent kinase 4 or 6, and this complex phosphorylates and activates genes whose products regulate the G1/S transition of the cell cycle (52, 53). These data suggest that HDAC1 and DNMT3A exist in a common complex, which is associated with the OCT3/4 expression in CC cells (50).

Furthermore, the clinicopathological significance of OCT3/4 in cervical squamous cell carcinoma (CSCC) and its correlation with occurrence and prognosis were investigated using CSCC tissue and normal cervical tissue, finding that OCT3/4 expression was higher in CSCC tissue than in normal cervical tissue; in addition, the expression of OCT3/4 and SOX2 was significantly related to the Wnt signaling pathway (54). Wnt/β-canonical signaling plays an important role in the self-renewal, pluripotency, proliferation, and determination of the cellular fate of ESCs; it was found that Wnt/β-catenin signaling is one of the key pathways in the maintenance of CSC (e.g., in lung, colon, liver, and breast cancer) (55). Both biomarkers of ESC (SOX2 and OCT3/4) and the Wnt signal pathway (β-catenin) are activated in CSCC (54).

Additionally, it has been reported that SOX2 and OCT3/4 stem cell biomarkers could be used to predict radioresistance in patients with locally advanced CSCC (LACSCC). Shen et al. (56) evaluated the expression of OCT3/4 and SOX2 by immunohistochemistry in two groups: the radioresistant group and a group sensitive to radiation; they showed that the expression of SOX2 and OCT3/4 was higher in the group resistant to radiation than in the sensitive group. This suggested that the expression of SOX2 and OCT3/4 in tumor cells indicates radioresistance and is important an predictor of poor survival in patients with LACSCC (56). On the other hand, it was discovered that two pseudogenes of *OCT3/4*, that is, *OCT3/4-pg5* and *OCT3/4-pg1*, are transcribed in cancer (57). *OCT3/4-pg5* is transcribed in cells and cancer tissues, whereas *OCT3/4-pg1* is found only in cancerous tissues at a low level (57), while Hayashi et al. (58) found that it is overexpressed in gastric cancer when compared to its normal counterpart. The transcription of these pseudogenes in cancer samples suggests that they may play a role in the regulation of *OCT3/4* gene activity and carcinogenesis (57).

Previously, Mueller et al. (59) demonstrated that only OCT3/4B is present in cell lines of somatic tumors (colorectal carcinoma cells, thyroid carcinoma, cervical carcinoma, head and neck carcinoma, non–small cell lung carcinoma hepatoma, breast carcinoma, ovarian carcinoma, prostate carcinoma, neuroblastoma, glioblastoma, and melanoma) (59). Moreover, recent studies have demonstrated overexpression of OCT3/4A in patients with hepatocellular carcinoma in prostate cancer, lung cancer, hepatocellular carcinoma, and breast cancer and also *in vivo* models of prostate cancer (60–62).

To understand the biological functions of the two OCT3/4 isoforms in CC cells, Li et al. (46) evaluated sphere formation efficiency in stable cell lines: SiHa-OCT3/4A and SiHa-OCT3/4B and showed that SiHa-OCT3/4A cells had increased sphere formation capacity, whereas SiHa-OCT3/4B generated few or even no tumor spheres. This confirmed that OCT3/4A promoted tumor sphere formation in CC cell lines and that OCT3/4A was responsible for self-renewal of CSCs (46). In addition, overexpression of OCT3/4B in the CC SiHa cell line favors cell proliferation; tumorigenesis by inhibiting apoptosis; enhanced angiogenesis by positive regulation of CD34, VEGF, HIF-1α, and IL-6; tumor cell migration to the surrounding tissue through the upregulation of MMP2 and MMP9; and induction of EMT (46). Thus, both isoforms cooperate in diverse functions to regulate the progression of CC (Figure 1). Furthermore, it was observed that the reduction in the expression of OCT3/4B inhibited cell proliferation and cell migration, promoted cell apoptosis both *in vitro* and *in vivo*, and showed that OCT3/4B has functions as a novel tumor oncogene in CC, which may serve as an effective diagnostic biomarker and a potential therapeutic target in the treatment of CC (63).

### Oct3/4 and E6 and E7 Oncoproteins

During HR-HPV infection, the virus binds to the receptors on the target cell surface, resulting in its internalization, and subsequently, the viral DNA is released and transported to the cell nucleus (45, 64). After the viral DNA is integrated, it synthesizes the E6 and E7 oncoproteins that promote proliferation for TP53 degradation by E6 and pRB degradation by E7; it has been observed that the degradation of pRb leads to the overexpression of OCT3/4 and that the degradation of p53 leads to an increase in the expression of NANOG (65, 66), and it is well known that NANOG can directly bind to the *OCT3/4* gene promoter to induce its expression (67–69). Recently, it was observed that the E6 and E7 oncoproteins of HPV16 increase expression levels of a subset of stem cell marker genes, including OCT3/4, NANOG, and SOX2 both *in vivo* and *in vitro* and that cells expressing the E6 and E7 oncoprotein exhibit a greater self-renewal capacity (70, 71). These data suggest that the E6 and E7 oncoproteins increase the self-renewal of cancer cells.

## Conclusion

Because CC is one of the leading causes of death in women worldwide, it is important to understand the mechanisms involved in its development and progression. The data presented here demonstrate a role for OCT3/4 in cervical carcinogenesis. The high expression of OCT3/4 was associated with cancerous cells and tissues, and it was suggested that it has the ability to promote CC. Moreover, OCT3/4 has been suggested as an important biomarker of prognosis and resistance to chemotherapy and radiotherapy in patients with CC.

## Author Contributions

SC-P, YG-G, and JO-N collated the references and wrote this review. AL-M, ML-V, JO-N, and BI-A reviewed and edited the manuscript.

### Conflict of Interest

The authors declare that the research was conducted in the absence of any commercial or financial relationships that could be construed as a potential conflict of interest.
